# Supplementary material for: Shared Genetic Architectures between Coronary Artery Disease and Type 2 Diabetes Mellitus in East Asian and European Populations
Source: Biomedicines. 2024 Jun 3;12(6):1243. doi: 10.3390/biomedicines12061243 (PMC11201280; doi:10.3390/biomedicines12061243)
Supplement: Supplementary file 1 [file biomedicines-12-01243-s001.zip › Supplementary material.pdf]

Supplementary material

Data collecting and processing of Chinese cohort

Quality control of the validation study with PLINK 2.0 includes a call rate greater than 95%, Hardy-Weinberg equilibrium > 10−4, and ATP calling. Detection of uncertain kinships and ethnicity outliers was also performed as described in our previous study [3]. In the quality control step, Manual inspection of genotype clusters was first carried out for >55,000 variants that either (1) showed evidence of bad genotype clustering in exome array genotyping of over 9,000 subjects by collaborators17,55, or (2) had GenTrain score <0.8 or (3) had high missingness (>1%). A total of 4,550 markers were removed due to poor genotype clustering. Indiviudal-level QC was carried out with regard to duplication, gender mismatch, possible sample contamination and biological relatedness. We further employed a variant-level QC that removed SNPs with >2% missingness or violated Hardy–Weinberg equilibrium (P<1 × 10−5), or SNPs originally designed with the purpose of quality control. Details regarding the quality controls are given in the Supplementary Methods. After all quality control measures, 5,233 samples and 286,795 SNPs, of which 176,149 variants were monomorphic, remained in the data set and were subject to association analysis. We conducted GWAS in each cohort by a logistic regression model using PLINK v2 software (https://www.cog-genomics.org/plink/2.0). We included sex, age and top 5 principal components, constructed by genotype data, within as covariates.

Function annotation and definition of common pleiotropic loci

FUMA assigns the lead SNPs and additional independent SNPs in each genomic risk locus to their mapped genes (defined as candidate genes here) not only based on genomic position, but also on three kind of Function annotation method, namely, combined annotation dependent depletion (CADD) score, probability of regulatory functionality (Regulome DB score), and transcription/regulatory effects from chromatin states (Minimum chromatin state)[1-3]. The SNPs in high LD ($r^2 \geq 0.6$) with lead SNPs or independent SNPs and the distances among them less than 250Kb were selected as candidate SNPs. Those Candidate SNPs were further used for functional annotation. CADD scores the deleteriousness of an SNP by summarizing 63 Function annotations with a support vector machine, SNP with $CADD\ score \geq 12.37$ is more likely to be pathogenic[1]. Regulome DB, ranging from 1 to 7, assesses the possibility of transcription factor binding and gene expression being affecting by a SNP based on ENCODE and other sources. SNPs with $Regulome\ DB\ score \leq 2$ were defined as functional signals[2]. Minimum chromatin state can predict the accessibility of chromatin region, and was established by a multivariate hidden Markov model with 15 categorical states on the basis of 5 histone modification marks for 127 epigenomes[3]. A score of 8-15 indicates the closed chromatin state. On the 1-7 scale, lower score indicates more open chromatin states, suggesting the higher DNA regulatory potential of the genomic region where candidate SNP was located. For more detail, see Supplementary material.

Clinical characteristics of CAD cohort.

The clinical characteristics of the CAD cohort are summarized in Table S1. The median age was 63.81±8.01 years, with 1,650 (62%) of the cohort being female. 40% of samples had been

diagnosed with hypertension; 75% and 32% of CAD patients reported a history of CAD and T2DM. The median FBG and OGTT in CAD patients were 6.74±2.28 and 10.66±8.60 mmol/L, respectively. Significant results from the comparison between the two groups suggest the comorbidity between T2DM and CAD.

Table S1 Clinical characteristics of samples in CAD cohort

**Characteristic**	**Overall**, N = 2,647	**Controls**, N = 2,042	**CAD**, N = 605	**P-value**[1]
Age, years,Mean (SD)	63.81 (8.01)	63.62 (7.64)	64.44 (9.13)	0.061
Gender, n (%)				0.2
male	997 (38%)	755 (37%)	242 (40%)	
female	1,650 (62%)	1,287 (63%)	363 (60%)	
Hypertension, n (%)				<0.001
No	1,578 (60%)	1,362 (67%)	216 (36%)	
Yes	1,052 (40%)	668 (33%)	384 (64%)	
Self-report of CAD, n (%)				<0.001
No	2,128 (83%)	1,982 (99%)	146 (25%)	
Yes	450 (17%)	14 (0.7%)	436 (75%)	
Angina pectoris, n (%)				<0.001
No	2,376 (94%)	1,982 (100%)	394 (74%)	
Yes	141 (5.6%)	0 (0%)	141 (26%)	
Ischemic cardiomyopathy, n (%)				<0.001
No	2,446 (97%)	1,982 (100%)	464 (87%)	
Yes	71 (2.8%)	0 (0%)	71 (13%)	
Unknown	2	1	1	
Myocardial Infarction, n (%)				<0.001
No	2,560 (97%)	2,041 (100%)	519 (86%)	
Yes	85 (3.2%)	0 (0%)	85 (14%)	
FBG, mmol/L, Mean (SD)	6.18 (1.72)	6.01 (1.47)	6.74 (2.28)	<0.001

Characteristic	**Overall**, N = 2,647	**Controls,** N = 2,042	**CAD,** N = 605	**P-value**[1]
OGTT, mmol/L, Mean (SD)	8.97 (5.83)	8.46 (4.59)	10.66 (8.60)	<0.001
Self-report of T2DM, n (%)				<0.001
No	2,184 (83%)	1,772 (87%)	412 (68%)	
Yes	458 (17%)	268 (13%)	190 (32%)	
Antidiabetics, n (%)				<0.001
No	2,253 (85%)	1,817 (89%)	436 (72%)	
Yes	385 (15%)	219 (11%)	166 (28%)	
Unknown	9	6	3	

[1]Wilcoxon rank sum test; Pearson's Chi-squared test; Fisher's exact test

Clinical characteristics of T2DM cohort.

The clinical characteristics of the T2DM cohort are outlined in Table S2. With a median age of 58.97±8.70 years, the cohort comprised 1912 (64%) females. Hypertension was diagnosed in 36% of the samples, while 56% and 20% of T2DM patients reported a history of T2DM and CAD, respectively. For T2DM patients, the median FBG and OGTT were 7.97±2.46 and 14.21±8.28 mmol/L, respectively. Similar to the findings in the CAD cohort, significant results from the comparison between the two groups also indicate a notable comorbidity between T2DM and CAD.

Table S2 Clinical characteristics of samples in T2DM cohort

Characteristic	**Overall**, N = 2,995	**Controls**, N = 2,315	**T2DM**, N = 680	**p-value**[1]
Age, years, Mean (SD)	58.97 (8.70)	57.95 (8.35)	62.47 (8.96)	<0.001
Gender, n (%)				<0.001
male	1,083 (36%)	769 (33%)	314 (46%)	
female	1,912 (64%)	1,546 (67%)	366 (54%)	
FBG, mmol/L, Mean (SD)	6.06 (1.62)	5.50 (0.49)	7.97 (2.46)	<0.001
OGTT, mmol/L, Mean (SD)	8.50 (5.22)	6.82 (1.64)	14.21 (8.28)	<0.001
Self-report of T2DM, n (%)				<0.001
No	2,594 (87%)	2,296 (99%)	298 (44%)	
Yes	393 (13%)	19 (0.8%)	374 (56%)	

Characteristic	**Overall**, N = 2,995	**Controls**, N = 2,315	**T2DM**, N = 680	**p-value**[1]
Antidiabetics, n (%)				<0.001
No	2,668 (89%)	2,312 (100%)	356 (53%)	
Yes	318 (11%)	3 (0.1%)	315 (47%)	
Hypertension, n (%)				<0.001
No	1,911 (64%)	1,596 (69%)	315 (47%)	
Yes	1,065 (36%)	703 (31%)	362 (53%)	
Self-report of CAD, n (%)				<0.001
No	2,565 (89%)	2,042 (92%)	523 (80%)	
Yes	322 (11%)	189 (8.5%)	133 (20%)	
Angina pectoris, n (%)				<0.001
No	2,725 (97%)	2,133 (97%)	592 (94%)	
Yes	93 (3.3%)	57 (2.6%)	36 (5.7%)	
Unknown	2	2	0	
Ischemic cardiomyopathy, n (%)				0.045
No	2,768 (98%)	2,157 (98%)	611 (97%)	
Yes	50 (1.8%)	33 (1.5%)	17 (2.7%)	
Unknown	2	2	0	
Myocardial Infarction, n (%)				<0.001
No	2,940 (98%)	2,287 (99%)	653 (96%)	
Yes	53 (1.8%)	26 (1.1%)	27 (4.0%)	

[1]Wilcoxon rank sum test; Pearson's Chi-squared test; Fisher's exact test

Supplementary Reference

1 Kircher M, Witten DM, Jain P, O'Roak BJ, Cooper GM and Shendure J. A general framework for estimating the relative pathogenicity of human genetic variants. *Nat Genet* 2014; **46**: 310-5.

2 Boyle AP, Hong EL, Hariharan M, Cheng Y, Schaub MA, Kasowski M, et al.

Annotation of functional variation in personal genomes using RegulomeDB. *Genome Res* 2012; **22**: 1790-7.

3 Kundaje A, Meuleman W, Ernst J, Bilenky M, Yen A, Heravi-Moussavi A, et al. Integrative analysis of 111 reference human epigenomes. *Nature* 2015; **518**: 317-30.
